# Developing a JAK Inhibitor for Targeted Local Delivery: Ruxolitinib Cream

**DOI:** 10.3390/pharmaceutics13071044

**Published:** 2021-07-08

**Authors:** Paul Smith, Wenqing Yao, Stacey Shepard, Maryanne Covington, Jim Lee, Jennifer Lofland, Ahmad Naim, Trupti Sheth, Bhavnish Parikh, Swamy Yeleswaram

**Affiliations:** 1 Incyte Research Institute, 1801 Augustine Cut-Off, Wilmington, DE 19803, USA; shepard@incyte.com (S.S.); MCovington@incyte.com (M.C.); yeleswaram@incyte.com (S.Y.); 2 Incyte Corporation, 1801 Augustine Cut-Off, Wilmington, DE 19803, USA; WYao@incyte.com (W.Y.); jilee@incyte.com (J.L.); jlofland@incyte.com (J.L.); anaim@incyte.com (A.N.); tsheth@incyte.com (T.S.); bparikh@incyte.com (B.P.)

**Keywords:** Janus kinase, inflammation, atopic dermatitis, vitiligo, topical

## Abstract

Named after the two-faced Roman god of doorways, Janus kinases (JAKs) represent a class of tyrosine kinases. The JAK signaling pathway is pivotal for the downstream signaling of inflammatory cytokines, including interleukins, interferons, and multiple growth factors. This article provides an overview of the JAK pathway and signaling, its significance in immune-mediated dermatologic diseases and the development of a targeted, localized option of a selective JAK inhibitor, ruxolitinib cream. In the early 1990s, various discovery and clinical development programs were initiated to explore pharmaceutical inhibition of the JAK-STAT pathway. Incyte Corporation launched a strategy to identify molecules suitable for both topical as well as oral delivery. Ruxolitinib was designed as a molecule with low nanomolar potency selective for JAK1 and 2 enzymes, but without significant inhibition of non-JAK kinases, as well as physicochemical properties for both topical and oral administration. An oil-in-water emulsified ruxolitinib cream formulation was developed for topical application and was studied in multiple immune-mediated dermatologic diseases including psoriasis, alopecia areata, atopic dermatitis and vitiligo. Ruxolitinib cream represents a novel, JAK1/2 selective therapy that can be delivered directly to the skin to treat a number of cytokine-driven, inflammatory dermatoses.

## 1. Overview of the JAK-STAT Pathway

Named after the two-faced Roman god of doorways, Janus kinases (JAKs) represent a class of tyrosine kinases that contain two near-identical phosphate-transferring domains: a catalytic domain and a second autoregulatory domain. The JAK family includes JAK1, JAK2, JAK3 and TYK2 (tyrosine kinase 2) [1]. Ligand-mediated receptor binding and dimerization brings two JAKs into close proximity allowing transphosphorylation and activation. Activated JAKs subsequently phosphorylate and activate signal transducers and activators of transcription (STATs). The mammalian STATs family consists of seven members (STAT1, STAT2, STAT3, STAT4, STAT5A, STAT5B and STAT6). Following phosphorylation, STATs are translocated to the nucleus, and dimerize and bind specific regulatory sequences to activate or repress transcription of target genes [2]. Thus, the JAK-STAT cascade provides a direct mechanism to translate an extracellular signal into a transcriptional response. The JAK-STAT-mediated signaling pathway is ubiquitous among vertebrates and found in many other metazoans [3,4]. The JAK-STAT pathway is pivotal for the downstream signaling of inflammatory cytokines, including interleukins (ILs), interferons (IFNs) and multiple growth factors [5]. Overall, the selective use of JAKs by different receptors coupled to downstream STAT signal transduction results in an elegant mechanism to achieve exquisite in vivo specificity for more than 60 cytokines and growth factors [5].

## 2. JAK-Mediated Inflammation

An overwhelming body of evidence has established that JAK-dependent cytokines are major contributors to immunopathology and that blocking such cytokines can be beneficial in immune-mediated diseases [6]. For instance, IL-6 is a prototypic proinflammatory cytokine commonly overexpressed in many autoimmune and inflammatory diseases [7] and is a driver of acute-phase responses including induction of C-reactive protein and serum amyloid A leading to acute and chronic inflammation [8]. The efficacy of monoclonal antibodies that target IL-6 or its receptor in rheumatologic diseases confirms the criticality of this cytokine in immunopathogenesis [7].

Similarly, there are extensive data to support the pathogenic role of IL-12 and IL-23 in inflammatory bowel disease and psoriasis; the efficacy of ustekinumab, a monoclonal antibody targeting the p40 subunit of both cytokines, strongly supports this conclusion [9]. The overexpression of IL-4, IL-5 and IL-13 in allergic disease and the success of drugs that target these cytokines [10,11,12] provide a rationale for the potential utility of interfering with type I/II cytokine signaling in disorders such as asthma and atopic dermatitis (AD). Many other JAK-dependent cytokines have been shown, in various settings, to contribute to inflammatory diseases. These include but are not limited to IL-15, IL-21, IFN, granulocyte colony-stimulating factor and granulocyte-macrophage colony-stimulating factor [13].

It is well-established that type I/II cytokine receptors require JAKs to exert their effects, and that other receptor super families do not. The dependence of type I and type II cytokines on JAKs was established in a variety of genetic models, from mutagenized cell lines and knockout mice to humans with mutations [13,14,15]. Polymorphisms in JAK and STAT genes are associated with autoimmunity, and loss-of-function mutations cause immunodeficiency due to the inability of type I/II cytokines to transmit signals through their receptors [13,14,15]. More recent phosphoproteomic analysis established that for the IL-2 receptor, at least 90% of signaling is JAK dependent [16]. The criticality of this pathway to type I/II cytokine signaling was further evidence that interfering with the activity of JAKs could lead to a new class of immunomodulatory drugs [17,18], and uncovered some potential adverse effects of JAK blockade.

## 3. Development of JAK Inhibitors

The therapeutic potential of the JAK-STAT pathway led to the development of targeted inhibitors of JAK enzymes. In the early 1990s, Merck published a patent (WO 03/011285) for a JAK inhibitor; however, no clinical candidates were reported [19]. In addition, Pfizer disclosed CP-690,550 (subsequently named tofacitinib) as a JAK inhibitor clinical candidate for solid organ transplantation in 2003 and shifted its focus to rheumatoid arthritis in 2005 [20]. In addition, more companies, including Incyte Corporation and Cytopia, initiated discovery programs to identify JAK inhibitors. The discovery of the JAK2 V617F mutation in 2005 thrust the JAK family into the limelight. The V617F mutation in JAK2 is strongly associated with myeloproliferative neoplasms (MPNs), including myelofibrosis, polycythemia vera and essential thrombocythemia, occurring in nearly 100% of patients with polycythemia vera and over 75% of patients with essential thrombocythemia [21]. Soon after the discovery of the mutation, numerous biopharmaceutical companies announced leads and/or clinical candidates. Large pharmaceutical companies also joined the fray with AstraZeneca, Bristol Myers Squibb, Novartis and Eli Lilly and Company entering early-phase clinical development. Most of the programs were focused on JAK2 with an eye on MPN, with the sole exception of Pfizer, which was continuing to focus on rheumatoid arthritis.

From the outset, Incyte was active in progressing selective JAK inhibitors from a research aspiration into a clinical reality [22,23,24,25]. Incyte had seeded a discovery program in oncology. The initial focus was on multiple myeloma but with the discovery of V617F mutation in JAK2, attention shifted to MPN and myelofibrosis in particular. Yet, the utility of JAK inhibitors in the treatment of autoimmune diseases was recognized and a decision was made to seek one molecule for MPN and another molecule to be dedicated for autoimmune diseases. Further, it was felt that dermatologic diseases such as plaque psoriasis would benefit from topical application and that mild to moderate psoriasis, in particular, would demand a high therapeutic index, which is better served with local rather than systemic delivery. This early recognition led to a two-pronged strategy—to identify a molecule with a longer half-life and lower clearance for oral administration in MPN and another molecule with a shorter half-life and higher clearance for local delivery.

Incyte’s Medicinal Chemistry group, composed of chemists with decades of collective experience in identifying and developing selective high-quality molecules against challenging targets across multiple target classes, was well supported by biologists who performed appropriate primary, secondary, and tertiary assays for biological activity and pharmaceutical properties. After extensive efforts the team was able to identify attractive molecules. Multiple structural series (scaffolds) were evaluated in parallel. The first series yielded potent molecules but suffered from poor pharmaceutical properties. Specific structural modifications were made to improve solubility and other pharmacokinetic properties, which resulted in structurally distinct, azepine-based analogs. This series, however, suffered from weak potency in whole-blood assays owing to high protein binding. Through structure–activity relationship (SAR) studies on these early series, structural elements that imparted a desirable JAK1/2 selectivity profile were discovered. The extensive experience of the medicinal chemistry team was then leveraged in the design of new scaffolds that combined selectivity-conferring features with hinge-binding pharmacophores in an arrangement more likely to possess favorable physicochemical properties. Molecular docking was utilized in the evaluation of the designed scaffolds, and this was followed by SAR-guided lead-seeking efforts. Throughout the lead-seeking phase, selectivity against non-JAK kinases was given the highest priority. Rapid and iterative feedback from the biology and absorption, distribution, metabolism, and excretion (ADME) teams enabled the chemists to quickly discover improvements to the potency and pharmaceutical parameters of the new series. The pyrazole-substituted pyrrolo[2,3-d]pyrimidine series emerged as an attractive scaffold, and it provided molecules with excellent potency in biochemical, cell-based, and whole-blood assays as well as good selectivity and pharmacokinetic profiles.

Two molecules were identified from this series: a molecule with lower clearance and longer half-life for oral administration in MPN, and another molecule, INCB018424 (subsequently named ruxolitinib), that exhibited excellent permeability and solubility but a shorter half-life in animal models, which was selected for local delivery. However, the molecule selected for MPN had a suboptimal nonclinical toxicology profile and was, therefore, discontinued. Ruxolitinib, on the other hand, had cleared nonclinical toxicology studies, and a decision was made to develop ruxolitinib for both oral and local delivery.

## 4. Targeted Application to the Skin

**Figure 1:**
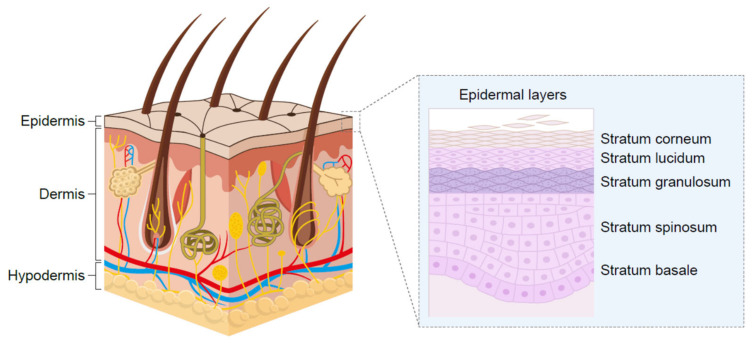
Structure of human skin.

Skin, the largest organ, serves as a physiological barrier to environmental agents including bacteria, fungi and chemicals while preventing the loss of fluids and solutes from the internal environment. The stratum corneum is the outermost layer of the epidermis (Figure 1) [26] and is about 20 µm thick, highly keratinized and provides a first line of barrier function. The layer below, the living epidermis, is five- to six-fold thicker than the stratum corneum and hydrophobic in nature. Sitting beneath the epidermis, the dermis region of the skin comprises roughly half the total skin thickness, is made up of connective tissue and is well vascularized. The hypodermis, or the layer beneath the dermis, is made up of adipocytes and plays a role in energy homeostasis.

For distribution into the dermis following topical administration, the drug should possess (a) adequate solubility to achieve high enough concentrations to drive the first-order diffusion across the keratinized stratum corneum as well as (b) adequate permeability to rapidly establish a dynamic equilibrium. Besides permeability and solubility, the molecule needs to have the right hydrophilic-lipophilic balance to partition out of the hydrophobic epidermis and into the hydrophilic dermis. These three physicochemical properties—aqueous solubility, permeability and hydrophilic-lipophilic balance—are critical to achieving pharmacologically active concentrations in the skin. Lastly, the molecule should have a high clearance and short intrinsic half-life to enable quick elimination of any fraction of the dose that reaches systemic circulation.

The molecular parameters that influence these physicochemical properties are as follows: (a) molecular weight—rule of thumb threshold is 500 daltons, with molecular weight of >500 generally considered not conducive to skin delivery, (b) clogP—a measure of lipophilicity, and (c) aqueous solubility–approximately 1 mg/mL, with or without low concentrations of solubilizing agents [27,28,29,30]. Ruxolitinib satisfied the above requirements and hence was considered for topical administration. Another parameter, polar surface area, which is affected by both molecular weight and the presence of heteroatoms, is inversely correlated to passive permeability. Therefore, this parameter can be useful in the design of molecules intended for topical administration. The clogP influences the hydrophilic-lipophilic balance as well as propensity for nonspecific binding in skin tissue. Whereas some degree of binding to the epidermis is beneficial in enabling a depot formation, higher and more avid binding can significantly decrease the partitioning out of that layer, which in turn can result in very low concentrations in the dermis. Presence of more than three aromatic rings in a molecule may decrease solubility and/or increase the probability of phototoxicity [30,31]. Through Incyte’s medicinal discovery research, ruxolitinib was discovered to possess the criteria for molecular weight, lipophilicity, aqueous solubility, polar surface area, and number of aromatic rings.

The monophosphate salt form of ruxolitinib was selected for development for both oral and local delivery, based on superior aqueous solubility compared to free base. An initial assessment of solubility of ruxolitinib phosphate in various individual solvents and binary solvent systems suitable for topical delivery demonstrated higher solubility in aqueous systems (1–10 mg/mL) and poor solubility in nonaqueous vehicles (<0.2 mg/mL).

Both oil-in-water and water-in-oil emulsions were considered, and based on physicochemical evaluation, solubilized oil-in-water emulsion was selected for further development.

As part of formulation development, topical formulations of ruxolitinib were evaluated for transport across human cadaver skin using Franz diffusion cells with the cream formulation showing the highest flux [32]. The highest concentration or strength of ruxolitinib 1.5% *w/w* was selected, to prevent any precipitation during storage. The list of all excipients in the final formulation is shown in Table 1.

A preclinical study was conducted to compare the pharmacokinetics and skin distribution of ruxolitinib following topical versus oral delivery [32]. Following oral administration in minipigs, the area under the plasma concentration time curve was approximately 31-fold higher than that observed following local administration of ruxolitinib cream 1.5% twice daily. The average total dermis concentration of ruxolitinib at steady state after topical administration was 507-fold higher versus that following oral dosing, demonstrating a substantial skin-targeting advantage with topical application. The concentration of unbound ruxolitinib in the dermis after topical application was thought to result in sustained and near-complete inhibition of JAK-STAT signaling in this tissue. In contrast, only partial inhibition of downstream signaling was thought to occur after oral dosing. This preclinical study indicated that the dermal distribution profile of ruxolitinib cream should maximize the efficacy in the skin while minimizing the potential for deleterious systemic effects.

Short-term stability studies indicated overall good physical stability of the emulsion as well as no hint of any chemical instability. Subsequent longer-term stability studies have confirmed this early observation. There is a slight drop in viscosity of the cream at 40 °C, but that change did not affect the application onto the skin. Once clinical development was initiated, anecdotal reports from study investigators and patients indicated a highly desirable feel to the cream. This same formulation was used in pivotal toxicology studies and clinical development programs.

## 5. Inflammatory Skin Diseases

The treatment of most inflammatory dermatoses involves the administration of medications directly to the affected sites. Local delivery can deliver higher levels of drug to the inflamed sites and reduce the potential side effects of systemic administration of many anti-inflammatory compounds. Many skin conditions have autoimmune or inflammation-mediated pathogenesis that may benefit from medications administered locally rather than systemically (e.g., AD, psoriasis and vitiligo).

**Figure 2:**
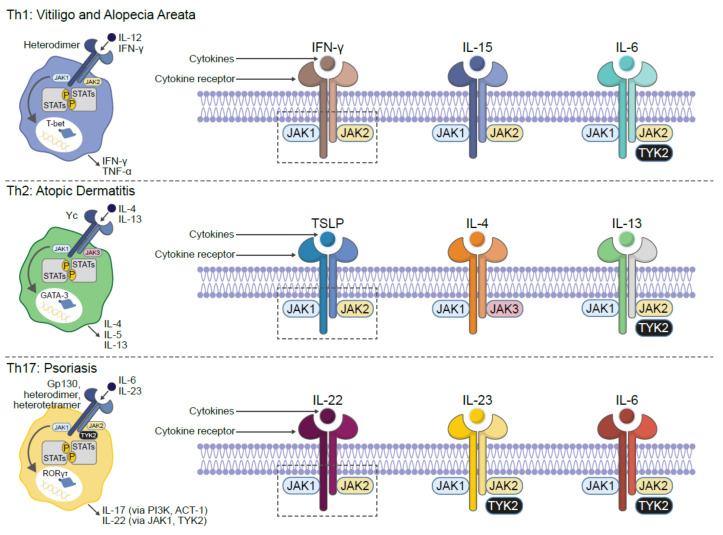
JAK-dependent inflammatory cytokines are implicated in the pathogenesis of multiple dermatologic diseases. ACT 1: NF-κB activator 1; GATA: GATA binding protein-3; IFN: interferon; IL: interleukin; JAK: Janus kinase; PI3K: phosphoinositide-3-kinase; ROR: retinoid-related orphan receptor; STAT: signal transducer and activator of transcription; TYK: tyrosine kinase. Figure adapted from reference [5].

**Figure 3:**
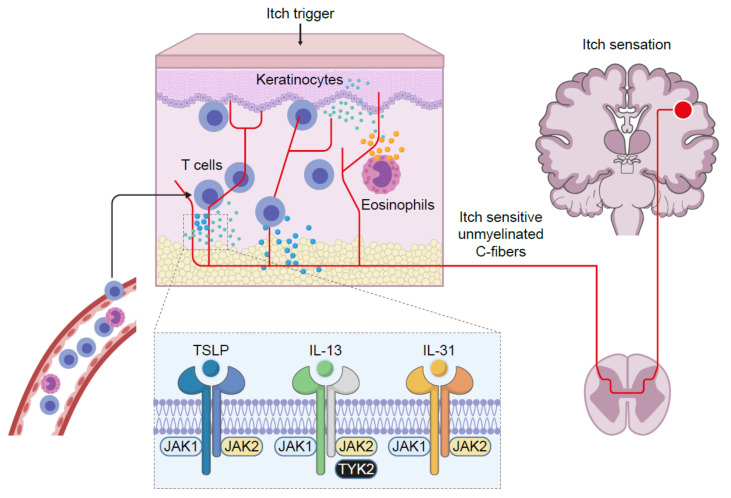
JAK-dependent signaling significantly contributes to chronic itch. IL: interleukin; JAK: Janus kinase; TSLP: thymic stromal lymphopoietin; TYK: tyrosine kinase.

AD is a life-long, chronic, inflammatory heterogenous skin disease, which manifests as recurrent eczematous lesions along with persistent itch that negatively affect quality of life through sleep disturbances, anxiety and depression. The prevalence of AD is up to 20% in children and 10% in adults, with rates varying geographically [5]. Skin barrier disruption resulting in allergen exposure facilitates the development of AD [33]. JAK1-mediated Th2 cytokines IL-4 and IL-13 negatively affect skin barrier integrity by inhibiting the expression of filaggrin, loricrin and involucrin, resulting in destabilization of tight junctions [34,35]. Lesional skin is characterized by cellular infiltrate primarily consisting of CD4+ T cells and overexpression of inflammatory Th2 cytokines (IL-4, IL-13, IL-31) [36]. Crucially, the cytokines IL-4, IL-13 and IL-31 require JAK-STAT downstream signaling for their biological function [37,38,39] (Figure 2). Furthermore, AD skin transcriptome analysis revealed increased JAK1 expression in lesional and non-lesional tissue [40]. In addition to inflammation, pruritic cytokines, thymic stromal lymphopoietin (TSLP) and IL-31 use downstream JAK1 and JAK2 signaling [39,41] (Figure 3). Neuronal IL-4Rα acting via JAK1 signaling also significantly contributes to chronic itch [42].

Psoriasis is a chronic, autoimmune, erythematosquamous dermatosis condition, characterized by skin lesions, which are red and scaling. Psoriasis has a prevalence of approximately 2–3% across the world [5]. Within psoriatic lesions, infiltrating autoreactive lymphocytes, mainly represented by IL-17-producing Th17 cells, Th1 and Th22 subsets, release IL-17, IFNγ, IL-22 and tumor necrosis factor alpha(TNFα) to potentiate the inflammatory milieu and perpetuate the pathogenic cycle [43,44]. Multiple critical immune mediators are inextricably linked to the JAK-STAT signaling pathway. For example, within psoriatic skin, dermal dendritic cells and macrophages produce IL-23, which once bound to its cognate receptor, uses JAK1/2/TYK2 signaling to promote Th17 cell expansion and survival [45]. Th17 and γδ T cells are the primary source of IL-22 in psoriatic skin, and this cytokine triggers reduced differentiation, increased proliferation and acanthosis in keratinocytes [46]. IL-22 binds to its IL-10R2 and IL-22R1 heterodimeric cell surface receptor coupled to JAK1/TYK2 and STAT3 signaling [43,47]. The anti-IL-22 monoclonal antibody fezakinumab (ILV-094) was clinically explored for safety and tolerability in a small phase 1 clinical trial (NCT00563524) of psoriasis patients; however, no peer-reviewed manuscript was published, and subsequent development was discontinued. Within the inflamed psoriatic lesion microenvironment, other cytokines, such as IL-6 and IL-21, can enhance IL-17 production from Th17 cells in a JAK-STAT-dependent manner [48,49].

Vitiligo is a chronic, autoimmune depigmenting disorder that results from destruction of melanocytes, causing white spots on the affected skin. The disease can be stigmatized by society, resulting in a significant impact to a patient’s quality of life. The prevalence of vitiligo is approximately 0.5–2% across the world [5]. Antimelanocyte CD8+ T cells in the blood and skin correlate with disease severity, and lesional CD8+ T cells in vitro induce melanocyte apoptosis in unaffected skin [50,51]. These data support the rationale that autoimmune cytotoxic T lymphocytes are directly responsible for melanocyte destruction in human vitiligo. Expression analysis reveals an infiltrating Th1-type autoreactive CD8+ IFNγ-specific signature [51,52]. Transcriptome analysis on the skin and blood of patients with vitiligo revealed IFNγ-induced chemokines CXCL10 and CXCL9 were increased [53,54]. Furthermore, serum CXCL10 levels were associated with the Vitiligo Area Scoring Index (VASI) of patients with progressive vitiligo, suggesting that the CXCL10/CXCR3 axis mediates T cell recruitment into the skin of progressive vitiligo. Given the apparent critical role of IFNγ in driving vitiligo inflammation and its downstream signaling dependent on the JAK1-JAK2 heterodimer, it has also been found that intense and diffuse JAK1 expression is more present within vitiliginous skin compared with healthy tissue [55,56,57]. Tissue resident memory T cells (TRM) reside in peripheral, non-lymphoid tissues, and represent a new subset of memory T lymphocytes that provide localized protective immunity in tissues [58,59]. Skin TRM express tissue-specific homing antigens, including cutaneous lymphocyte antigen (CLA) and CCR8 as well as skin retention markers, including CD103 and CD69 [60]. Autoreactive TRM cells secreting IFNγ have been described in lesional vitiligo skin and are responsible for disease relapse [61,62,63]. IL-15 is a member of the IL-2 cytokine family and potentiates survival, maturation, and cytotoxicity of TRM cells [64,65]. Patients with vitiligo have significantly higher serum IL-15 levels compared to healthy controls, and this positively correlated to vitiligo disease severity [66]. In lymphocytes, IL-15 binding to the IL-2/15Rβγ heterodimer receptor requires JAK1/JAK3 downstream signaling, resulting in STAT3/5 phosphorylation [67]. Additionally, in a preclinical mouse model of vitiligo, antibody-mediated inhibition of IL-15 signaling provided long-lasting repigmentation [63].

Alopecia areata is an autoimmune disease resulting in partial or complete nonscarring hair loss, with a global prevalence of approximately 2% [68]. Early symptoms are typically characterized by small, well-defined patches of hair loss on the scalp or beard that may spontaneously resolve with time; however, subsequent relapses frequently occur. Multiple lines of evidence have demonstrated that alopecia areata pathogenesis is autoimmune in nature, with loss of immune privilege and associated T cell infiltration selectively attacking growth at the hair follicle (i.e., anagen phase) [69,70,71,72]. Global transcriptional profiling analyses of affected skin identified expression signatures indicative of cytotoxic T cell infiltration, such as increased production of IFNγ and γ-chain (γc) cytokines, including IL-15 [72,73]. Similar to IFNγ, IL-15 enhances innate and self-reactive memory T cell immunity, including autoimmune disease pathogenesis [73,74], and is JAK1 dependent [75].

## 6. Clinical Studies with Ruxolitinib Cream

Ruxolitinib cream has been studied in multiple dermatologic diseases including psoriasis, alopecia areata, AD and vitiligo. In a proof-of-concept study in individuals with psoriasis [76], patients were dosed with vehicle, 0.5% or 1.0% of ruxolitinib cream once a day or 1.5% twice a day for 28 days. Both the 1% and 1.5% cream improved lesion thickness, erythema and scaling and reduced lesion area compared to vehicle. Ruxolitinib cream was well tolerated with few mild adverse effects [76].

A phase 2 study was conducted that examined the safety and efficacy of 1.5% ruxolitinib cream in patients with alopecia areata who had at least 25% hair loss by Severity of Alopecia Tool score [77]. The results revealed that there was no significant difference in hair regrowth based on 50% improvement in Severity of Alopecia Tool scores between patients receiving 1.5% ruxolitinib cream and vehicle in part B. There were no significant safety issues with ruxolitinib cream [77].

For AD, Incyte conducted two phase 3, randomized studies (TRuE-AD1 [NCT03745638] and TRuE-AD2 [NCT03745651]) of adult and adolescent patients with AD. Significantly more patients treated with either ruxolitinib cream regimen achieved the primary endpoint of Investigator Global Assessment (IGA) treatment success at week 8 (44.7% and 52.6% for 0.75% and 1.5% ruxolitinib, respectively) versus vehicle (11.5%; all *p* < 0.0001). Eczema Area and Severity Index (EASI) 75 (75% improvement in EASI score from baseline) at week 8 was achieved by 53.8% and 62.0% of patients who applied 0.75% ruxolitinib and 1.5% ruxolitinib, respectively, versus 19.7% on vehicle (all *p* < 0.0001). Substantially greater itch reduction was observed within 12 h of first ruxolitinib cream application (mean change from baseline: –0.4 and –0.5 for 0.75% ruxolitinib and 1.5% ruxolitinib) versus vehicle (–0.1; all *p* < 0.02). At week 8, more patients who applied ruxolitinib cream achieved a four-point improvement from baseline on the Itch Numeric Rating Scale (Itch NRS4) (41.5% and 51.5% for 0.75% ruxolitinib and 1.5% ruxolitinib, respectively) versus vehicle (15.8%; all *p* < 0.0001). No adverse events indicative of systemic activity of ruxolitinib were observed. Overall, no ruxolitinib-related serious adverse events were reported [78].

For vitiligo, a multicenter, randomized, double-blind, phase 2 study was conducted, which examined two doses, 0.75% and 1.5%, of ruxolitinib cream versus vehicle. The primary endpoint was the proportion of patients achieving a 50% or higher improvement from baseline in the Facial Vitiligo Area Severity Index (F-VASI50) at week 24. The results revealed that F-VASI50 at week 24 was reached by significantly more patients given ruxolitinib cream at 1.5% twice daily and 1.5% once daily than those who were treated with vehicle. All treatment-related adverse events were mild to moderate in severity and similar across treatment groups [79]. Currently, Incyte is conducting two phase 3 studies (NCT04052425; NCT04057573) to evaluate the efficacy and safety of ruxolitinib cream in adolescent and adult participants with nonsegmental vitiligo.

## 7. Conclusions

The JAK-STAT pathway is pivotal for the downstream signaling of inflammatory cytokines, including interleukins, interferons and multiple growth factors [5]. JAK inhibition is important in dermatologic conditions [37]. Ruxolitinib was discovered as a molecule with appropriate molecular properties for topical application, achieving local targeting of proinflammatory and pruritic cytokines through selective JAK inhibition. Ruxolitinib cream appears to be both efficacious and safe in the treatment of cytokine-driven dermatologic conditions.

## Figures and Tables

**Table 1:** Ruxolitinib topical composition [32].

Topical Ingredient	
Propylene glycol	Cetyl alcohol
Methylparaben	Stearyl alcohol
Propylparaben	Dimethicone 360
Xanthan gum	Medium-chain triglycerides
Light mineral oil	Edetate disodium
Glyceryl stearate	Polyethylene glycol 200
Polysorbate 20	Phenoxyethanol
Petrolatum, white	Water
Ruxolitinib phosphate (free base equivalent)	

## Data Availability

Not applicable.
